# Reproductive period and preclinical cerebrospinal fluid markers for Alzheimer disease: a 25-year study

**DOI:** 10.1097/GME.0000000000001816

**Published:** 2021-07-02

**Authors:** Jenna Najar, Tore Hällström, Anna Zettergren, Lena Johansson, Erik Joas, Madeleine Mellqvist Fässberg, Henrik Zetterberg, Kaj Blennow, Silke Kern, Ingmar Skoog

**Affiliations:** 1 Neuropsychiatric Epidemiology Unit, Department of Psychiatry and Neurochemistry, Institute of Neuroscience and Physiology, Sahlgrenska Academy, Centre for Ageing and Health (AGECAP) at the University of Gothenburg, Mölndal, Sweden; 2 Region Västra Götaland, Sahlgrenska University Hospital, Psychiatry, Cognition and Old Age Psychiatry Clinic, Gothenburg, Sweden; 3 Department of Psychiatry and Neurochemistry, Institute of Neuroscience and Physiology, The Sahlgrenska Academy at the University of Gothenburg, Mölndal, Sweden; 4 Clinical Neurochemistry Laboratory, Sahlgrenska University Hospital, Mölndal, Sweden; 5 Department of Neurodegenerative Disease, UCL Institute of Neurology, London, United Kingdom; 6 UK Dementia Research Institute at UCL, London, United Kingdom.

**Keywords:** Alzheimer disease, Cerebrospinal fluid markers, Menopause, Reproductive period, Risk factors in epidemiology

## Abstract

**Objective:**

The aim of the study was to examine the association between reproductive period, as an indicator of endogenous estrogen, and levels of cerebrospinal fluid (CSF) biomarkers for Alzheimer disease (AD).

**Methods:**

A population-based sample of women from Gothenburg, Sweden was followed from 1968 to 1994 (*N* = 75). All women had natural menopause and were free from dementia. Information on reproductive period (age at menarche to age at menopause) was obtained from interviews from 1968 to 1980. Lumbar puncture was performed from 1992 to 1994 and CSF levels of Aβ42, Aβ40, P-tau, and T-tau were measured with immunochemical methods. Linear regression models adjusted for potential confounders were used to analyze the relationship between reproductive period and CSF biomarkers for AD.

**Results:**

Longer reproductive period was associated with lower levels of Aβ42 (β = −19.2, *P* *=* 0.01), higher levels of P-tau (β = 0.03, *P* *=* 0.01), and lower ratio of Aβ42/Aβ40 (β = −0.02, *P* *=* 0.01), while no association was observed for T-tau (β = 0.01, *P* *=* 0.46). In separate analyses, examining the different components of reproductive period, earlier age at menarche was associated higher levels of P-tau (β = −0.07, *P* *=* 0.031) and lower ratio of Aβ42/Aβ40 (β = 0.05, *P* *=* 0.021), whereas no association was observed with Aβ42 (β = 31.1, *P* *=* 0.11) and T-tau (β = −0.001, *P* *=* 0.98). Furthermore, no association was observed between age at menopause and CSF biomarkers for AD.

**Conclusions:**

Our findings suggest that longer exposure to endogenous estrogen may be associated with increased levels of AD biomarkers in the preclinical phase of AD. These findings, however, need to be confirmed in larger samples.

***Video Summary:***.

The lifetime risk of Alzheimer disease (AD) is greater in women compared to men, especially after the age of 80 years.^1,2^ Estrogen is suggested as a possible explanation.^3^ In basic science studies, estrogen exposure is reported to be neuroprotective.^4-7^ The effect of hormone therapy (HT) on dementia risk in epidemiological studies and randomized controlled trials is, however, less conclusive; HT has been associated with both increased^8,9^ and reduced^8,10-12^ risk of dementia, whereas some studies found no association.^13,14^

Results from studies examining endogenous estrogens on dementia risk are also conflicting. Previous studies have reported an association between both higher^15,16^ and lower^17^ levels of estradiol in serum and risk of cognitive decline and dementia, whereas other studies report no associations.^18-20^ Furthermore, studies examining the reproductive period as an indicator of endogenous estrogen report that both longer^21,22^ and shorter^12,23,24^ reproductive periods have been associated with cognitive decline, dementia, and AD risk, whereas another study found no association between reproductive period and risk of dementia.^25^

Moreover, several neuroimaging studies have reported conflicting results on the effect of estrogen exposure (measured as HT,^26-29^ indicators of endogenous estrogen,^30^ menopausal transition,^31^ or levels of estradiol in serum)^16^ on biomarkers for dementia (particularly AD). Thus far, studies examining the association between estrogens and biomarkers for AD in cerebrospinal fluid (CSF) are, however, lacking. In CSF, brain pathology of AD is reflected by lower levels of amyloid-β 1-42 (Aβ42), lower ratio of Aβ42 and amyloid-β 1-40 (Aβ40), and higher levels of hyperphosphorylated tau (P-tau), whereas greater intensity of neurodegeneration correlate with higher CSF levels of total tau (T-tau).^32^

We aimed to examine the long-term associations between reproductive period, as an indicator of endogenous estrogen, and levels of biomarkers for AD in CSF (Aβ42, P-tau, T-tau, and ratio Aβ42/Aβ40) in a population-based sample of women free from dementia and with natural menopause, followed over 25 years.

## METHODS

### Study population

As part of the Gothenburg H70 Birth Cohort Studies, we examined levels of CSF biomarkers for AD in the Prospective Population Study of Women from Gothenburg, Sweden. From 1968 to 1969, 1,462 women (participation rate 90%) born in 1908, 1914, 1918, 1922, and 1930 (mean age 46 years) participated in a health examination.^33^ The women were systematically selected from the Swedish Population Registry based on specific birth dates to yield a representative sample at the ages studied.^33^ Re-examinations occurred from 1974 to 1975, 1980 to 1981, and 1992 to 1994, as described in more detail previously.^22,34^

In the follow-up from 1992 to 1994, women aged 70 years or older (ie, born 1908, 1914, 1918, and 1922) were invited to an extensive neuropsychiatric examination (*n* = 590 women, participation rate 68%), and 88 consented to a lumbar puncture (LP). Participants and nonparticipants in the LP were similar regarding baseline blood pressure, body mass index, smoking status, cardiovascular disease, depressive symptoms, report of longstanding stress, and Mini-Mental State Examination score at the 1992 to 1994 examination as reported previously.^34^ In addition, there were no differences between those who participated and those who did not participate in the LP examination regarding length of reproductive period (*P* = 0.4), age at menarche (*P* = 0.3) and menopause (*P* = 0.7), number of pregnancies (*P* = 0.3), months of breastfeeding (*P* = 0.2), miscarriages (none vs one or more; *P* = 0.6), HT (previous vs never user; *P* = 0.6), and *APOE ε4* status (*P* = 0.6). LP participants were, however, younger, had higher education, developed dementia less often after follow-up examinations, and had a lower 5-year mortality rate compared to nonparticipants, as previously described.^34^ Furthermore, a higher proportion of LP participants compared to nonparticipants had used oral contraceptives (*P* = 0.04).

Seven women were excluded due to lack of data on reproductive period, four due to dementia, and two due to surgical menopause, leaving 75 dementia-free women with natural menopause (born 1908 [*n* = 2], 1914 [*n* = 6], 1918 [*n* = 32], and 1922 [*n* = 35]).

The Ethics Committee for Medical Research at the University of Gothenburg approved the study and all participants gave informed consent to participate according to the Declaration of Helsinki.

### Assessment of CSF biomarkers

LPs were conducted between January 1992 and March 1994. LPs were performed through the L3/L4 interspace and CSF samples of 12 mL were collected and gently mixed to avoid gradient effects.^35^ To eliminate cells and other insoluble materials, the samples were centrifuged at 2,000 *g* for 10 minutes and stored at −80°C in 1-mL polypropylene vials until analyses.^36,37^ Sandwich enzyme-linked immunosorbent assays were used to determine levels of Aβ42, Aβ40, P-tau, and T-tau.^36-38^

### Reproductive period

Information on age at menarche and menopause, and type of menopause (natural, premature, or surgical) were obtained from semistructured interviews during 1968 to 1969, 1974 to 1975, and 1980 to 1981, ensuring coverage of the entire reproductive period of all included birth cohorts.^22^ Age at menarche was defined as first menstruation, and menopause as last menstruation after 1 year without menstruation.^22^ Age at menarche and menopause were used as continuous variables in all analyses. Length of reproductive period was defined as the time from age at menarche to age at menopause.

### Diagnosis of dementia

The diagnosis of dementia was based on the *Diagnostic and Statistical Manual of Mental disorders, Third Edition—Revised* (*DSM-III-R*) criteria,^39^ using information from semistructured neuropsychiatric examinations and close informant interviews.^40^ Dementia was only used for exclusion in the study (*n* = 4).

### Potential confounders

Information on covariates were obtained at baseline examination (defined as first examination after menopause) from 1968 to 1969 (mean age 53 years), 1974 to 1975 (mean age 54 years), or 1980 to 1981 (mean age 58 years), either through semi-structured interviews (education, number of pregnancies, months of breastfeeding, number of miscarriages, use of HT and oral contraceptives, angina pectoris, stress, physical activity, and smoking) or through health examinations (hypertension, waist-hip ratio, electrocardiography [ECG]) or by a combination of these (hypertension, myocardial infarction).^22^ Education level was categorized into four groups based on years of education (group 1 had ≤6 years, group 2 had 7-9 years, group 3 had 10-12 years, and group 4 had >12 years of education). The last given information on number of pregnancies (defined as the sum of children and miscarriages in 1968-1969, and number of pregnancies in 1974-1975 and 1980-1981) and months of breastfeeding was used to ensure coverage of all events.^22^ Number of pregnancies and months of breastfeeding were used as continuous variables in all analyses.^22^ Number of miscarriages was used as a dichotomous variable (none vs one or more). The women reported duration of oral contraceptives use. Oral contraceptives was used as a continuous variable and as a dichotomous variable (previous vs never user) to deal with the high proportion of zeros (ie, women who never used oral contraceptives).^41^ Physical activity was based on the Saltin-Grimby Physical Activity Level Scale including four groups: group 1 was inactive, group 2 engaged in light physical activity for a minimum of 4 hours/week, group 3 performed regular physical exercise for at least 2 hours/week, and group 4 had regular to intense physical exercise.^42^ Physical activity was further dichotomized as inactive (group 1) and active (groups 2-4).^43^ Hypertension was defined as systolic blood pressure of 160 mmHg or greater or diastolic blood pressure of 95 mmHg or greater, or taking antihypertensive medication.^22^ Smoking was divided into current/former smoker and nonsmoker. Waist-hip-ratio was defined as the ratio between waist and hip circumference. The diagnosis of ischemic heart disease was defined as having angina pectoris, myocardial infarction, and/or ECG changes, as described previously.^22^ The diagnosis of myocardial infarction was based on information from interviews, medical records, and death certificates. Angina pectoris was diagnosed according to Rose criteria. ECG was performed on all participants during rest, and ischemic heart disease was diagnosed according to Minnesota codes 1.1-2, 4.1, 5.1-2 (in the absence of 3.1), 6.1, or 7.1.^22^ Psychological stress was defined as frequent or constant stress symptoms, for example, tension, nervousness, and sleep disturbance (≥1 month) in relation to circumstances in everyday life. From 1992 to 1994, the women reported duration of HT use, which was used as a continuous variable and as a dichotomous variable (previous vs never user) to deal with the high proportion of zeros (ie, women who never used HT).^41^

### Statistical analysis

All analyses were conducted in R (version 4.0.0) using the R stats and ggplot2 packages. T-tau and P-tau were natural log transformed to improve symmetry of the distributions. Demographic and health characteristics are presented as numbers, mean and median values, standard deviations (SDs), interquartile ranges, minimum (min) and maximum (max) values, and percentages. Linear regression models were used to analyze the relation between length of reproductive period and levels of CSF biomarkers for AD (Aβ42, P-tau, T-tau, and ratio Aβ42/Aβ40), presented as β-coefficients, *P* values (two-sided), standard error, and *R*^2^ in two different models. Model 1 included reproductive period and birth year as a covariate. The selection of covariates for Model 2 was done in three steps. First, we chose covariates of importance for AD based on the literature. Second, all covariates (education, number of pregnancies, months of breastfeeding, number of miscarriages, oral contraceptives, HT, stress, physical activity, smoking, waist-hip ratio, hypertension, angina pectoris, and myocardial infarction) were analyzed in relation to the outcomes (Aβ42, ratio Aβ42/Aβ40, P-tau, and T-tau), respectively in a model including the covariate of interest, reproductive period, and birth year. Third, the covariates that were associated with the outcome at a *P* value threshold less than 0.3 were included in Model 2. Covariates selected for Model 2 of Aβ42 were birth year, oral contraceptives, waist-hip ratio, and education; for Model 2 of P-tau were birth year, psychological stress, smoking status, and education; for Model 2 of T-tau were birth year and psychological stress; and for Model 2 of Aβ42/Aβ40 were birth year, waist-hip ratio, and psychological stress.

A sensitivity analysis was performed excluding major outliers (ie, 3 × interquartile range) from P-tau (*n* = 1), to eliminate the possibility that the association was driven by extreme values.

We also analyzed the two components of reproductive period (age at menarche and menopause) separately in relation to CSF biomarkers of AD in crude linear regression models adjusted for birth year.

Finally, we tested the correlation between birth year and age at LP from 1992 to 1994, to examine whether the adjustment for birth year in our models (Model 1 and Model 2) was sufficient to minimize the risk that the observed associations were driven by age at LP from 1992 to 1994.

## RESULTS

Table 1 shows demographic factors and health characteristics in women with a natural menopause and with data on CSF biomarkers for AD (*n* = 75). The median age at baseline was 52 years (min, max, 46, 60 years). The mean time from baseline to LP was 20.2 years (SD 3.8). Median age at LP was 74 years (min, max, 70, 85 years). The mean length of reproductive period for women with natural menopause was 35.4 years (SD 3.8, range 25.0-43.0). The mean age at menarche and menopause for women with natural menopause was 14 years (SD 1.5) and 49.4 years (SD 3.5, range 38.0-57.0), respectively. Compared to those excluded (*n* = 13), women included in the study (*n* = 75) had lower baseline age (age 53 [SD 3.4] years vs 61.5 [SD 9.5] years; *P* < 0.001), whereas no differences were observed regarding educational level, smoking status, physical activity, waist-hip ratio, hypertension, ischemic heart disease, psychological stress, or *APOE ε4* carriership.

**TABLE 1:** Demographic and health characteristics of participants

Characteristics	All participants (*n* = 75)
Age at baseline (yr), median (min, max)	52 (46, 60)
Age at LP (yr), median (min, max)	74 (70, 85)
Reproductive period (yr), mean (SD)	35 (3.8)
Age at menarche (yr), mean (SD)	14 (1.5)
Age at menopause (yr), mean (SD)	49 (3.5)
Pregnancies, number, median (min, max)	2 (0, 7)
Miscarriages, number, median (min, max)	0 (0, 4)
Breastfeeding^*a*^ (mo), median (min, max)	9 (0, 56)
Oral contraceptives, previous user % (cases/total number)	12 (9/75)
Hormone treatment, previous user % (cases/total number)	6 (4/71)
Education, >6 yr, % (cases/total number)	37 (28/75)
Smoking, current or previous % (cases/total number)	40 (30/75)
Physical activity, active % (cases/total number)	81 (61/75)
Waist-hip ratio, mean (SD)	0.7 (0.1)
Hypertension, % (cases/total number)	19 (14/75)
Ischemic heart disease, % (cases/total number)	27 (20/75)
Psychological stress, frequent or constant stress % (cases/total number)	39 (29/75)
*APOE ε4* carriership, % (cases/total number)	31 (16/52)
Aβ42 (pg/mL), mean (SD)	820 (28)
Aβ40 (pg/mL), median ± IQR (min, max)	9998 ± 5089 (5417, 21331)
Aβ42/Aβ40 ratio, mean (SD)	0.1 (0.03)
P-tau (pg/mL), median ± IQR (min, max)	20 ± 10 (10, 80)
T-tau (pg/mL), median ± IQR (min, max)	286 ± 204 (83, 1200)

Median (±IQR) are reported for variables with skewed distributions. Mean (SD) are reported for variables with a normal distribution.

Aβ40, free 40 amino acid Aβ peptides; Aβ42, free 42 amino acid Aβ peptides; *APOE ε4*, ε*4* allele of *apolipoprotein E* gene; IQR, interquartile range; LP, lumbar puncture; P-tau, phosphorylated tau; SD, standard deviation; T-tau, total tau.

a *n* = 73.

**FIG. 1:**
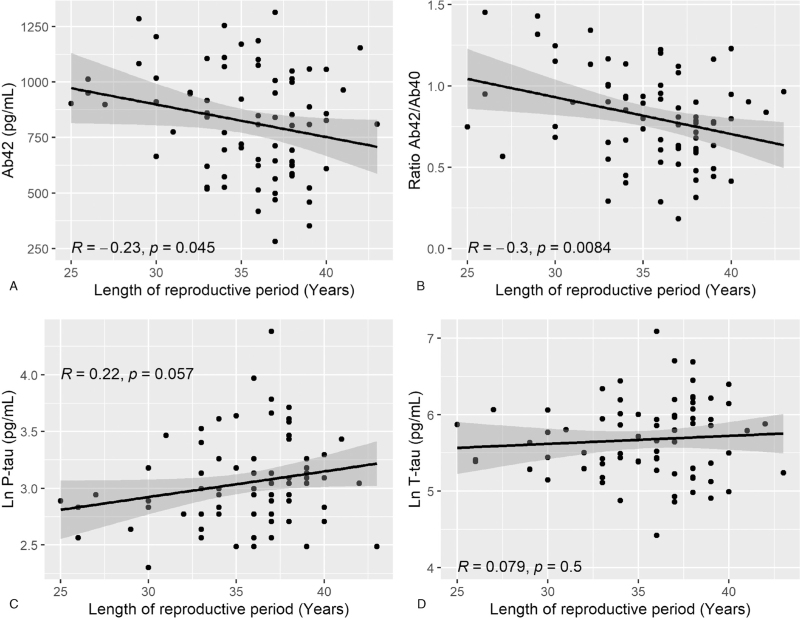
Reproductive period in relation to cerebrospinal fluid (CSF) biomarkers for Alzheimer disease. Scatterplot of length of reproductive period in relation to cerebrospinal fluid (CSF) levels of Aβ42 **(A)**, ratio of Aβ42/Aβ40 **(B)**, P-tau **(C)**, and T-tau **(D)**. Length of reproductive period is shown in years. Levels of CSF Aβ42, P-tau, and T-tau are shown in pictogram (pg)/milliliter (mL). P-tau and T-tau were natural log transformed (Ln) to improve symmetry of the distributions. Pearson correlation coefficient is reported as *R* and *P* values. Aβ42, free 42 amino acid Aβ peptides; Aβ42/Aβ40, ratio of free 42 amino acid Aβ peptides and free 40 amino acid Aβ peptides; P-tau, phosphorylated tau; T-tau, total tau.

In Model 1, longer reproductive period was associated with lower levels of Aβ42 (β = −14.5, *R*^2^ = 0.04, and *P* *=* 0.048) and lower ratio of Aβ42/Aβ40 (β = −0.02, *R*^2^ = 0.1, and *P* *=* 0.0084) (Table 2, Fig. 1). In Model 2, longer reproductive period was associated with lower levels of Aβ42 (β = −19.2, *R*^2^ = 0.1, and *P* *=* 0.011), lower ratio of Aβ42/Aβ40 (β = −0.02, *R*^2^ = 0.1, and *P* *=* 0.011), and higher levels of P-tau (β = 0.03, *R*^2^ = 0.1, and *P* *=* 0.011). No association was observed between length of reproductive period and levels of T-tau (Table 2). Scatter plots of the relationship between length of reproductive period and CSF biomarkers for AD are shown in Figure 1.

**TABLE 2:** Association between reproductive period and levels of cerebrospinal fluid biomarkers for Alzheimer disease (*N* = 75) in two different models

	Model 1			Model 2		
	SE	β-Coefficient	*P*	SE	β-Coefficient	*P*
Aβ42	7.2	−14.5	0.048	7.5	−19.2	0.01
P-Tau	0.01	0.02	0.060	0.01	0.03	0.011
T-Tau	0.01	0.01	0.52	0.01	0.01	0.46
Aβ42/Aβ40	0.008	−0.02	0.0084	0.008	−0.02	0.011

Length of reproductive period is reported as per 1 year. Aβ42, P-Tau, and T-Tau are reported as pg/mL. Model 1: Included reproductive period and birth year. Model 2: Reproductive period in relation to Aβ42: birth year, oral contraceptives, waist-hip ratio, and education; P-tau: birth year, psychological stress, smoking status, and education; T-tau: birth year and psychological stress; Aβ42/Aβ40: birth year, waist-hip ratio, and psychological stress.

Aβ42, free 42 amino acid Aβ peptides; Aβ42/Aβ40, ratio of free 42 amino acid Aβ peptides and free 40 amino acid Aβ peptides; P-tau, phosphorylated tau; SE, standard error; T-tau, total tau.

In sensitivity analyses, where we removed one major outlier from P-tau, length of reproductive period remained associated with levels of CSF P-tau in Model 2 (β = 0.03, *R*^2^ = 0.07, and *P* *=* 0.011).

**FIG. 2:**
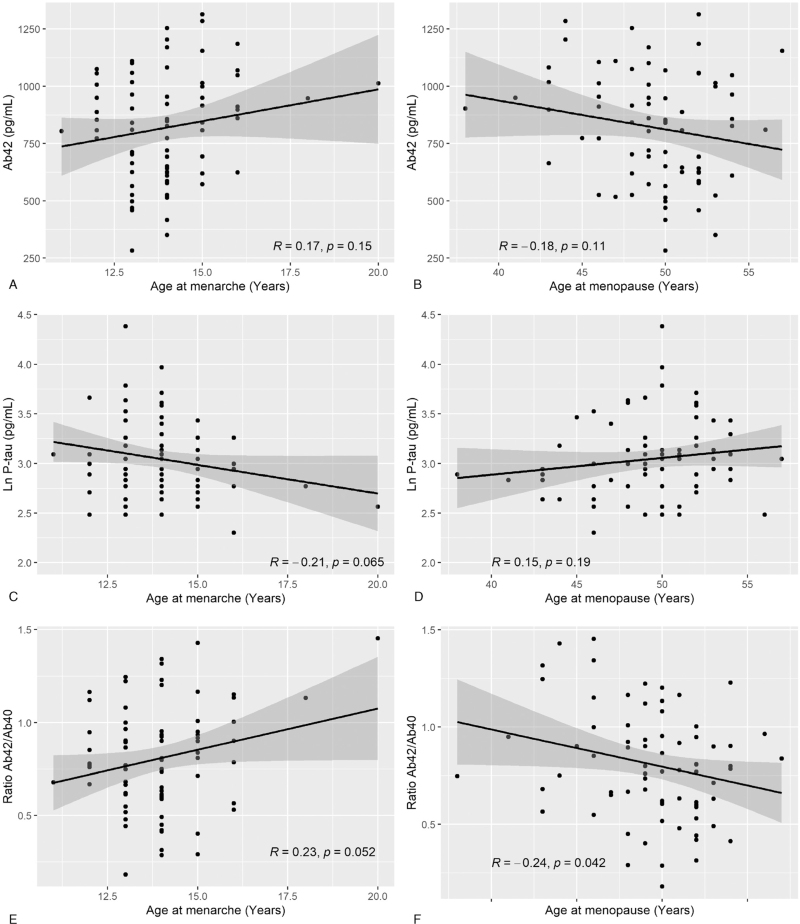
Age at menarche and menopause in relation to cerebrospinal fluid (CSF) biomarkers for Alzheimer disease. Scatterplots of age at menarche and menopause in relation to cerebrospinal fluid (CSF) levels of Aβ42 **(A, B)**, P-tau **(C, D)**, and ratio of Aβ42/Aβ40 **(E, F)**. Age at menarche and menopause are shown in years. Levels of CSF Aβ42 and P-tau are shown in pictogram (pg)/milliliter (mL). P-tau was natural log transformed (Ln) to improve symmetry of the distributions. Pearson correlation coefficient is reported as *R* and *P* values. Aβ42, free 42 amino acid Aβ peptides; Aβ42/Aβ40, ratio of free 42 amino acid Aβ peptides and free 40 amino acid Aβ peptides; P-tau, phosphorylated tau; T-tau, total tau.

In separate analyses, examining the different components of reproductive period in crude models adjusted for birth year, earlier age at menarche was associated with higher levels of P-tau (β = −0.07, and *P* *=* 0.031) and lower ratio of Aβ42/Aβ40 (β = 0.05, and *P* *=* 0.021), whereas no association was observed with Aβ42 (β = 31.1, and *P* *=* 0.11) and T-tau (β = −0.001, and *P* *=* 0.98). Furthermore, no association was observed between age at menopause and CSF biomarkers for AD (Aβ42: β = −12.0, and *P* *=* 0.13, P-tau: β = 0.01, and *P* *=* 0.25, T-tau: β = 0.01, and *P* *=* 0.49, Aβ42/Aβ40: β = −0.02, and *P* *=* 0.058). Scatter plots of the relationship between age at menarche and menopause and CSF biomarkers for AD, respectively are shown in Figure 2.

Finally, we found a strong correlation between age at LP and birth year (*R* = −0.99 *P* = 4 × 10^−116^). Thus, the inclusion of birth year in our models (Model 1 and Model 2) reduces the possibility that the observed associations were driven by different ages at LP from 1992 to 1994.

## DISCUSSION

In a population-based sample of women with natural menopause and free from dementia followed over 25 years, we found that longer reproductive period was associated with preclinical CSF markers of AD (lower levels of Aβ42, lower ratio of Aβ42/Aβ40, and higher levels of P-tau). We, however, found no associations between length of reproductive period and levels of CSF T-tau.

Our finding that longer reproductive period was associated with preclinical biomarkers for AD is in line with results from the same study reporting that longer reproductive period was associated with increased risk for dementia and a clinical diagnosis of AD, in women followed over 44 years, from 1968 to 2012.^22^ Further support comes from the Rotterdam Study reporting an increased risk of dementia in women with higher serum estradiol levels and longer reproductive period.^15,21^ Moreover, our findings are also consistent with results from neuroimaging studies. The UK Biobank reported that women with longer reproductive period had reduced total brain volume on MRI, compared to women with shorter period.^30^ In addition, the Rotterdam Study reported that higher total estradiol levels were associated with smaller hippocampal volumes in women.^16^ To the best of our knowledge, this is the first study to examine the association between reproductive period, as an indicator of endogenous estrogen, and CSF biomarkers of AD.

It needs to be emphasized that all women in this study were free from dementia. The process of AD is suggested to start 20 to 30 years before the clinical symptoms appear, with lowering levels of CSF Aβ42 as the first manifestation, whereas increased levels of tau occur later.^44^ Our findings suggest a negative effect of longer exposure to endogenous estrogen on CSF biomarkers for AD in the preclinical phase of AD. Thus, our results do not support the estrogen hypothesis, proposing a neuroprotective effect of estrogen. It should, however, be noted that the estrogen hypothesis is based mainly on studies examining exposure of exogenous estrogen (ie, estrogen from outside the body) on neuronal health and dementia risk.^45,46^ In fact, the majority of studies on endogenous estrogen and cognitive decline or risk of dementia report a negative effect of longer exposure, or no association.^45^ Also, reproductive period reflects long-term exposure to endogenous estrogen, whereas exogenous estrogen from HT for menopausal symptoms includes significantly higher levels of estrogen, but during a limited period. We had information on use of HT from the examination from 1992 to 1994, but only four women had used this treatment. Thus, we did not have the statistical power to analyze the relationships between HT and CSF biomarkers for AD. We can therefore not exclude the possibility that HT is related to CSF biomarkers for AD. Moreover, events contributing to fluctuations in levels of endogenous estrogens also involve variation in levels of other hormones, such as progesterone, follicle-stimulating hormone, and luteinizing hormone. Therefore, the effect of length of reproductive period on CSF biomarkers for AD in our study could be attributable to other hormones than estrogen.

Our findings that earlier menarche is associated with higher CSF levels of P-tau and lower ratio of Aβ42/Aβ40 are in line with our findings of reproductive period. We did not find a significant association between age at menopause and CSF biomarkers for AD. The associations were, however, similar to that of reproductive period as the estimates showed that later age at menopause was associated with lower levels of Aβ42, higher levels of P-tau, and lower ratio of Aβ42/Aβ40. Thus, considering our previous findings that the effect of reproductive period on dementia and AD was mainly driven by age at menopause,^22^ and that the estimates were similar for reproductive period and age at menopause, lack of significant associations between age at menopause and levels of CSF biomarkers for AD in the present study may be due to low statistical power.

Moreover, our finding that reproductive period was associated with levels of P-tau, but not to T-tau, suggests that the effects observed probably occurred at a preclinical stage of AD, before general neurodegeneration.^47,48^ P-tau is generally believed to be a more specific marker for AD pathology than T-tau, which is more related to unspecific neurodegeneration.^47,48^ Our findings therefore suggest that reproductive period is more related to AD pathology than to general neurodegeneration. Considering our relatively small sample size, it is, however, possible that low statistical power may also explain the lack of association between reproductive period and levels of T-tau.

Strengths of the study are the population-based sample, the long observation period, and the possibility to assess menopausal age close to the actual event. Several limitations should also be addressed. First, the sample size was relatively small. Some analyses may, therefore, be underpowered to detect small differences between groups. Second, cumulative attrition is a problem in long-term follow-up studies and those performing LP constituted only 10% of the eligible sample.^34^ In addition, considering that LP participants were younger, had higher education, developed dementia less often after follow-up examination, and had a lower 5-year mortality compared to nonparticipants,^34^ our sample could be regarded as healthier than the general population. Third, we only had information on CSF biomarkers for AD from the follow-up examination from 1992 to 1994. Fourth, we cannot exclude that there might be some recall biases. Although age at menopause was assessed close to the actual event, age at menarche was assessed several decades after it occurred. Fifth, even though we adjusted for number of pregnancies, months of breastfeeding, and number of miscarriages that contribute to fluctuations in levels of endogenous estrogen during the reproductive period, other factors affecting levels of endogenous estrogen, such as length and regularity of the menstrual cycle, were not considered. This could affect the validity of the reproductive period as an indicator of endogenous estrogens. Sixth, we previously reported that longer reproductive period and later age at menopause were related to increased mortality rate,^22^ which could affect the associations. Seventh, the study comprises Caucasian women from Gothenburg, Sweden, thus limiting the possibility to generalize our results to other populations. It is possible that differences in genetic composition and living conditions between populations in different geographical and cultural contexts may influence the association between reproductive period and CSF biomarkers of AD.

## CONCLUSIONS

In a population-based sample of women free from dementia and with natural menopause followed over 25 years, we found that longer reproductive period was associated with CSF biomarkers for AD, which may suggest that longer exposure to endogenous estrogen is associated with increased levels of AD biomarkers in the preclinical phase of AD. These results add to the understanding of the association between indicators of endogenous estrogen and AD. This may be one explanation for the higher life-time risk of AD in women compared to men. The findings, however, need to be confirmed in larger samples.

## Supplementary Material

Supplemental Digital Content
